# Identification of 27 Novel Variants in Genes *COL4A3, COL4A4*, and *COL4A5* in Lithuanian Families With Alport Syndrome

**DOI:** 10.3389/fmed.2022.859521

**Published:** 2022-03-28

**Authors:** Agne Cerkauskaite, Judy Savige, Karolina Janonyte, Ieva Jeremiciute, Marius Miglinas, Edita Kazenaite, Arvydas Laurinavicius, Rasa Strupaite-Sileikiene, Vija Vainutiene, Birute Burnyte, Augustina Jankauskiene, Arndt Rolfs, Peter Bauer, Sabine Schröder, Rimante Cerkauskiene

**Affiliations:** ^1^Institute of Biomedical Sciences, Faculty of Medicine, Vilnius University, Vilnius, Lithuania; ^2^Department of Medicine (Melbourne Health and Northern Health), Royal Melbourne Hospital, The University of Melbourne, Parkville, VIC, Australia; ^3^Faculty of Medicine, Vilnius University, Vilnius, Lithuania; ^4^Institute of Clinical Medicine, Faculty of Medicine, Vilnius University, Vilnius, Lithuania; ^5^Centre of Ear, Nose and Throat Diseases, Vilnius University Hospital Santaros Klinikos, Vilnius, Lithuania; ^6^Albrecht Kossel Institute for Neuroregeneration, University of Rostock, Rostock, Germany; ^7^CENTOGENE GmbH, Rostock, Germany

**Keywords:** Alport syndrome, *COL4A3*, *COL4A4*, *COL4A5* variants, novel, digenic inheritance, genotype-phenotype correlation

## Abstract

**Introduction:**

Alport syndrome (AS) is an inherited disorder characterized by hematuria, proteinuria, and kidney function impairment, and frequently associated with extrarenal manifestations. Pathogenic variants in *COL4A5* usually cause X-linked Alport syndrome (XLAS), whereas those in the *COL4A3* or *COL4A4* genes are associated with autosomal dominant (AD) or recessive (AR) inheritance. To date, more than 3000 different disease-causing variants in *COL4A5, COL4A3*, and *COL4A4* have been identified. The aim of this study was to evaluate the clinical and genetic spectrum of individuals with novel, pathogenic or likely pathogenic variants in the *COL4A3-A5* genes in a previously unstudied cohort.

**Methods:**

In this study molecular analysis by next generation sequencing (NGS) was performed on individuals from a Lithuanian cohort, with suspected AS. The presence of AS was assessed by reviewing clinical evidence of hematuria, proteinuria, chronic kidney disease (CKD), kidney failure (KF), a family history of AS or persistent hematuria, and specific histological lesions in the kidney biopsy such as thinning or lamellation of the glomerular basement membrane (GBM). Clinical, genetic, laboratory, and pathology data were reviewed. The novelty of the *COL4A3-A5* variants was confirmed in the genetic variant databases (Centogene, Franklin, ClinVar, Varsome, InterVar). Only undescribed variants were included in this study.

**Results:**

Molecular testing of 171 suspected individuals led to the detection of 99 individuals with 44 disease causing variants including 27, previously undescribed changes, with the frequency of 9/27 (33,3%) in genes *COL4A5, COL4A3* and *COL4A4* equally. Three individuals were determined as having digenic AS causing variants: one in *COL4A3* and *COL4A4*, two in *COL4A4* and *COL4A5*. The most prevalent alterations in genes *COL4A3-5* were missense variants (*n* = 19), while splice site, frameshift, unknown variant and stop codon changes were detected more in genes *COL4A4* and *COL4A5* and accounted for 3, 3, 1 and 1 of all novel variants, respectively.

**Conclusion:**

Genotype-phenotype correlation analysis suggested that some variants demonstrated intra-familial phenotypic variability. These novel variants represented more than half of all the variants found in a cohort of 171 individuals from 109 unrelated families who underwent testing. Our study expands the knowledge of the genetic and phenotypic spectrum for AS.

## Introduction

Alport syndrome (AS) is a progressive hereditary glomerular disease characterized by hematuria, proteinuria and progressive kidney function impairment with the prevalence of at least one in 2000 for the X-linked form and one in 100 for autosomal dominant (AD) inheritance (1). It is responsible for about 1–2% of all cases with kidney failure (KF) (2). AS can also be associated with extrarenal manifestations, including bilateral sensorineural hearing loss and specific ocular lesions (3).

AS is caused by pathogenic variants in the *COL4A3, COL4A4*, and *COL4A5* genes encoding type IV collagen α3, α4, and α5 chains, respectively. Pathogenic variants in *COL4A5* usually cause X-linked Alport syndrome (XLAS), while alterations in *COL4A3* and *COL4A4* genes are associated with autosomal dominant AS (ADAS) or autosomal recessive AS (ARAS) (4–6). There is also evidence of digenic inheritance in AS due to the transmission of variants in two of the three genes in addition to the classic Mendelian inheritance (7).

The type and position of AS pathogenic variants such as missense, nonsense, splice site or frameshift rearrangements in *COL4A3-A5* may lead to a variable kidney disease course with a different age at KF onset and manifestation of ocular and hearing abnormalities (8–13). Several studies have demonstrated a significant improvement in kidney function in AS individuals after early initiation and long-term treatment with angiotensin converting enzyme inhibitors (ACEi) (14–16). Therefore, early diagnosis of AS in individuals with persistent hematuria and proteinuria has a significant impact on prognosis. Molecular genetic testing of type IV collagen genes might hence provide information essential for diagnosis, prognosis, genetic counseling and treatment options (17). Previous papers have declared that a pathogenic variant was the best evidence for the diagnosis of AS (1). However, interpretation of these variants may be complicated due to the incomplete or conflicting evidence of pathogenicity. A recent consensus statement recently indicated that clinical features and family history of kidney failure or persistent hematuria, together with the identified genetic variant, are the strongest evidence for the diagnosis of AS (17).

To date, more than 3,000 different pathogenic variants in the *COL4A3, COL4A4*, and *COL4A5* genes are recorded (4). Many phenotypes of AS have been identified in a high number of individuals, but it appears that many possible variants of these genes are still unknown. A genetic diagnosis is essential to treatment and prognosis, and therefore the number of identified disease-causing variants and their clinical phenotype must be increased. In this study we report the results of an extensive mutational analysis and the genotype-phenotype correlations in a sizable Lithuanian cohort of 171 individuals that led to the identification of 27 novel variants in the type IV collagen genes.

## Study Participants and Methods

The study was approved by the Vilnius Regional Biomedical Research Ethics Committee of Lithuania (BioAlport, No 158200-16-857-367). All participants in this study signed a consent form regarding the interview and likely uses of their data. Consent from individuals under 18 years old was obtained from their parents.

All individuals were clinically examined by adult or pediatric nephrologists. The study cohort included the individuals treated at Vilnius University Hospital Santaros Klinikos (VUH SK) in < city>Vilnius < /city>, Lithuania from 2016 to 2021. Their age ranged from 2 months to 82 years old. Clinical diagnoses of individuals were made by a nephrologist based on clinical manifestations, laboratory analysis, such as hematuria, proteinuria and creatinine levels, and a family history of persistent hematuria or AS. Kidney histopathological data collection included light microscopy, immunofluorescence, and electron microscopy examination of biopsy tissues. All individuals with high-grade suspicion for AS that met the eligibility criteria (Table 1) provided a blood sample DNA extraction. This was applied onto a Dried Blood Spot DBS-filtercard, called CentoCard^®^ and sent to Centogene laboratory, in Rostock, Germany.

**Table 1:** Diagnostic features and criteria for suspicion of AS in our study.

Clinical features (persistent permanent microscopic hematuria, proteinuria, stage of chronic kidney disease based on eGFR (estimated glomerular filtration rate, using EPI-CKD formula) as follows: stage I (eGFR >90 mL/min/1.73 m^2^); stage II (eGFR 60–89 mL/min/1.73 m^2^); stage IIIa (eGFR 45–59 mL/min/1.73 m^2^); stage IIIb (eGFR 30–44 mL/min/1.73 m^2^); stage IV (eGFR 15–29 mL/min/1.73 m^2^); stage V (eGFR <15 mL/min/1.73 m^2^), bilateral sensorineural hearing loss, anterior lenticonus etc.)
Kidney biopsy (thinning, thickening, splitting or lamination of GBM, foot process effacement, FSGS)
Positive family history for clinical features of specific clinical criteria (positive for individuals with the symptoms above)
Positive family history of AS (currently diagnosed AS in family)
Incidental genetic diagnosis of AS searching the different diseases (ex. ADPKD, IgA nephropathy, nephronophthisis and etc.)

*GBM, glomerular basement membrane; FSGS, focal segmental glomerulosclerosis; ADPKD, autosomal dominant polycystic kidney disease*.

The genetic testing was performed by next generation sequencing (NGS) technologies in this laboratory. Genomic DNA was enzymatically fragmented, and regions of interest were enriched using DNA capture probes, targeted against the coding regions, and known pathogenic/likely pathogenic variants of panel genes. The libraries were then sequenced on an Illumina platform. The entire coding region of the *COL4A3, COL4A4*, and *COL4A5* genes, including 10 bp of flanking intronic sequences, were amplified and sequenced. Missing fragments were completed using classical Sanger sequencing to achieve 100% coverage of all genes of this panel. Data analysis, including alignment to the hg19 human reference genome (Genome Reference Consortium GRCh37), variant calling, and annotation was performed using validated software from Centogene. Data screening and bioinformatics analysis were performed depending on the targeted gene capture and high-throughput sequencing from DNA samples. The *in silico* prediction of the damaging effects of each identified variant was assessed using MutationTaster, PolyPhen-2 and SIFT. Splicing variants were analyzed by Human Splicing Finder (http://www.umd.be/HSF3/index.html) in addition to MutationTaster. Pathogenic variants which could not be classified with either of the *in silico* tools were declared as not applicable (N/a). Only previously undescribed in the literature variants, were included in this study. Genetic variants were checked and compared in five databases—Centogene, Franklin, ClinVar, InterVar and Varsome (last review in December 2021), and therefore classified into four categories due to their pathogenicity using The American College of Medical Genetics and Genomics (ACMG) criteria—pathogenic (class 1), likely pathogenic (class 2), variant with unknown significance (VUS, class 3) and likely benign variant/polymorphism (class 4). Most of the novel variants were submitted into ClinVar Database (accession SUB11112191 (SCV002098105 - SCV002098129), however in this study there was a partial non-availability of raw data due to the time period restriction imposed by Centogene laboratory on stored data (please see Data availability section).

Individuals with hetero/hemizygous pathogenic variants in *COL4A5 (1)* compound heterozygous or homozygous variants in *COL4A3* or *COL4A4 (2)*, singular heterozygous variants in *COL4A3* or *COL4A4 (3)*, or digenic variants in two of the following genes *COL4A3/A4/A5 (4)* were named as having XLAS, ARAS, ADAS, or digenic AS, respectively. Although there is still some debate about naming the individuals with heterozygous *COL4A3/A4* variants with ADAS, thin basement membrane nephropathy (TBMN) or carriers of autosomal recessive AS, in this study we chose to use the term ADAS following the Kashtan et al. proposed unified classification of AS in 2018 (13).

Individuals with a molecular diagnosis of AS were referred to an ophthalmologist where specific evaluation of the eyes was performed, including slit-lamp examination for corneal abnormalities, ophthalmoscopy for perimacular fleck retinopathy, retinoscopy for lenticonus and Optical Coherence Tomography (OCT) for temporal retinal thinning. In cases of ocular changes anterior segment and fundus photography were performed. Audiograms for screening bilateral hearing abnormalities were performed in most of the individuals. Family members with high index of suspicion for AS were referred for molecular analysis and consultation with a geneticist for further evaluation.

## Results

Molecular analysis of all exons in the *COL4A3, COL4A4*, and *COL4A5* genes of 171 individuals from 109 different families led to the detection of 44 genetic variants (in 51 families, 99 individuals), including 27 novel changes in 51 individuals (41% males and 59% females) from 26 unrelated families. To be noted, one *COL4A3* (c.520G>A (p.Gly174Arg) VCV000447175) and four *COL4A4* variants (c.594+1G>A VCV000438704.2; c.2756A>G (p.Glu919Gly) VCV000599099; c.3044G>A (Gly1015Glu) VCV000557146; c.2347G>A (p.Gly783Arg) VCV000252464) were reported in the ClinVar database by other laboratories, however no clinical data of the individuals with these variants were available. Our main aim was to describe genotype—phenotype correlations in previously undescribed variants.

Age at diagnosis ranged from 1 to 65 years (median age was 26.1 years) of the individuals with novel variants. Clinical data of screened individuals with an established genetic diagnosis are listed in detail in Supplementary Tables 1–3.

The frequency of the novel variants in each group of three *COL4A5, COL4A3*, and *COL4A4* genes was 33.3% (9/27) equally. Three individuals had compound heterozygous pathogenic variants in *COL4A3* or *COL4A4* genes, while no homozygous variants in these genes were detected. Three individuals were determined as having digenic AS causing variants: one in *COL4A3* and *COL4A4*, and two in *COL4A4* and *COL4A5*. In this study, 35% (9/26) of the families had XLAS, 8% (2/26) had ARAS, 8% (3/26) had digenic disease and the majority 54% (14/26) had one heterozygous variant in *COL4A3* or *COL4A4*, thus ADAS. Notably, some individuals with digenic or autosomal recessive AS were from the same families with XLAS or ADAS.

All individuals had microscopic hematuria with or without kidney failure. KF appeared in two males with likely pathogenic (according to ACMG, Varsome) variants in COL4A5, while ADAS in ARAS manifest to KF for one person in each group. A kidney biopsy had been performed on 22 individuals with AS, and all of them had particular histological abnormalities of the glomerular basement membrane (GBM).

Bilateral neurosensory hearing abnormalities and specific ophthalmologic lesions were seen in 12 and 9 individuals, respectively. Four out of 24 individuals (16.7%) had hearing abnormalities, while ocular abnormalities, including retinal flecks and retinal thinning, were found in six individuals (25%) with heterozygous COL4A3 and COL4A4 mutations. Bilateral hearing loss was seen in all three individuals with ARAS, while none had ocular abnormalities. Ocular changes of individuals with XLAS were found in four out of seven males (57.14%) and included anterior lenticonus, posterior subcapsular cataract and temporal retinal thinning. Hearing loss occurred in six males and one female with XLAS. Please see Table 2 and Supplementary Tables 1–3.

**Table 2:** Summary of demographic and clinical data of individuals with novel *COL4A3, COL4A4*, and *COL4A5* variants found in study.

	***COL4A3***		***COL4A4***		***COL4A5***	**Digenic inheritance**
	**ADAS**	**ARAS**	**ADAS**	**ARAS (or biallelic heterozygous)**		
Total number of individuals	8	2	16	2	14	3
Gender	F-4	F-2	F-8	F-1	F-13	F-2
	M-4	M-0	M-8	M-1	M-7	M-1
Age at diagnosis (range)	1–63	33	2-65	6–32	4–51	14–41
Number of individuals with KF (age at KF)	None	1	1	N/a	2	None
Number of individuals with ocular abnormalities	1	None	4	None	4	None
Number of individuals with hearing abnormalities	1	2	3	None	6	None
Number of individuals with kidney biopsy	5	2	3	2	7	2

*F, female; M, male; diff, different; KF, kidney failure; Ind, individual; ab., abnormalities; N/a, not applicable; N/d, no data; DIG, digenic; CKD, chronic kidney disease; FSGS, focal segmental glomerulosclerosis; pro, proteinuria; P, pathogenic; LP, likely pathogenic; VUS, variant with unknown significance; XLAS, X-linked Alport syndrome, AS, Alport syndrome, ARAS, autosomal recessive Alport syndrome; ADAS, autosomal dominant Alport syndrome*.

*Individuals with digenic Alport syndrome are included into digenic section despite the single novel variants in a particular gene*.

The most prevalent variants in genes *COL4A3-5* were missense changes (*n* = 19), 14 of which (74%) were glycine substitution. The splice site, frameshift, stop codon and unknown variants were more common in genes *COL4A4* and *COL4A5* and represented or 3, 3, 1 and 1 of all novel variants, respectively (Table 3).

**Table 3:** Summary of novel *COL4A3, COL4A4*, and *COL4A5* variants found in study.

**Type of pathogenic variant**	***COL4A3***	***COL4A4***	***COL4A5***
Total missense	9	6	4
Glycine substitution	7	3	4
Other missense variants	2	3	0
Stop codon	0	1	0
Splice site	0	1	2
Frame shift	0	0	3
Unknown variant	0	1	0

### COL4A5

Twenty-two individuals from 9 different families carried one variant in *COL4A5*. Summary of novel *COL4A5* variants found in a study can be seen in Table 4 and in more details in Supplementary Table 1. Seven males were diagnosed with XLAS. Fifteen individuals with heterozygous variants were females. Two females had digenic variants in genes *COL4A4* and *COL4A5*. Forty-one percent of the individuals with *COL4A5* changes were younger than 18 years old at the time at diagnosis. Nine novel variants were identified in the *COL4A5* gene (Table 4; Supplementary Table 1). According to the Centogene laboratory, three changes were classified as likely pathogenic, all three variants were described as disease causing or probably damaging by PolyPhen-2, while the rest of the variants were N/a. Two changes were judged as VUS while three of the variants were classified as disease causing. Five variants were considered as pathogenic using ACMG criteria in Varsome database, while InterVar four of these variants considered to be likely pathogenic and one as VUS.

**Table 4:** Summary of novel *COL4A5* variants found in a study (only heterozygous and hemizygous variants with X linked AS).

**Variant**	**No of ind**.	**No of M/F**	**No of diff. families**	**Age at diagnosis (range)**	**Pro**	**KF (age at KF)**	**No of ind. with ocular ab**.	**No of ind. with hearing ab**.	**Ind. with kidney biopsy**	**ACMG (ClinVar, Varsome, InterVar, Centogene, Franklin DB)**	**Our clinical interpretation on disease course**
**One hemizygous or heterozygous variant in** ***COL4A5*** **(XLAS)**											
c.3508G>C (p.Gly1170Arg)	3	M-1; F-2	1	11–43	+	N/a (but male has CKD IIIb)	None	None	1 (male with FSGS)	3-LP; 1-P; 1-N/A	Pathogenic
c.3106+1G>A	3	M-1; F-2	1	4–37	+	N/a	1	1	N/a	2-LP; 2-P; 1-N/A	Likely pathogenic
c.1417_1418del (p.Val473Glufs*3)	3	F-3	1	28–36	+	N/a (but female has CKD IIIa)	None	1	N/a	3-LP; 1-P; 1-N/A	Pathogenic (KF in family males)
c.347delC (p.Pro116Glnfs*39)	2	M-1; F-1	1	6–35	+	N/a	None	1	1 (lamellation of GBM)	2-LP; 2-P; 1-N/A	Likely pathogenic
c.883G>A (p.Gly295Ser)	1	M	1	22	+	N/a	None	None	1 (lamellation of GBM)	1-LP; 1-P; 1-N/A; 2-VUS	Likely benign course or late onset (normal eGFR)
c.2777G>T (p.Gly926Val)	3	M-2; F-1	1	24–47	+	2 males	2	2	1	1-LP; 1-P; 1-N/A; 2-VUS	Pathogenic
c.1374delinsTT (p.Pro459Serfs*6)	2	M-1; F-1	1	20–51	+	N/a	1	1	N/a	3-LP; 1-P; 1-N/A	Likely pathogenic or late onset form (eGFR for male is normal)
c.3554-2A>G	2	F-2	1	9–14	+	N/a	N/d	N/d	2 (FSGS)	3-LP; 1-P; 1-N/A	Likely pathogenic
c.466G>C (p.Gly156Arg)	1	F	1	15	+	N/a (CKD IIIb)	None	None	1 (FSGS)	1-P; 1-N/A; 3-VUS	Pathogenic

*No, number; F, female; M, male; diff, different; KF, kidney failure; Ind, individual; ab., abnormalities; N/a, not applicable; N/d, no data; DIG, digenic; CKD, chronic kidney disease; FSGS, focal segmental glomerulosclerosis; pro, proteinuria; P, pathogenic; LP, likely pathogenic; VUS, variant with unknown significance; XLAS, X-linked Alport syndrome, AS, Alport syndrome, ARAS, autosomal recessive Alport syndrome; ADAS, autosomal dominant Alport syndrome*.

Four *COL4A5* variants were identified as glycine substitutions. Two sequence variants were observed in *COL4A5* located at the splice donor site. The analysis of the pathogenic potential of these discovered variants using MutationTaster and Human Splicing Finder, showed functional loss of the affected protein caused by aberrant splicing. Three frame shift changes in *COL4A5* were also discovered (Table 4; Supplementary Table 1).

The clinical features are seen in Table 4 and Supplementary Table 1, while two individuals with digenic changes are described in digenic section. Proteinuria was discovered in 17 of 20 individuals. Two individuals with the same variant *COL4A5* p.Gly926Val reached KF at the age of 24 and 32, while eGFR < 60 ml/min/1.73 m^2^ was detected in 25% of individuals with *COL4A5* variants. Bilateral neurosensory hearing loss and ocular lesions were found in six and four individuals, respectively.

Our clinical interpretation of novel *COL4A5* variants was based on clinical course, family history, results of kidney biopsy, sex and age; and was listed in Table 4.

### COL4A3

Eleven individuals from eight different families had previously unreported variants in *COL4A3*. Summary of novel *COL4A3* variants found in a study can be seen in Table 5 and in more details in Supplementary Table 2. Eight with clinically diagnosed AS had one heterozygous variant, two individuals (twin sisters) both had compound heterozygous variants in *COL4A3*, and one had digenic variants in genes *COL4A3* and *COL4A4*. Altogether, nine novel variants were identified in *COL4A3* (Table 5; Supplementary Table 2). According to the Centogene laboratory, all nine novel variants were judged as variants with unknown significance by prediction tools (seven changes as probably damaging by PolyPhen-2 and eight as disease causing variants by MutationTaster, however one variant was described as polymorphism). To compare, Franklin genetic databases described two variants as pathogenic and eight variants as VUS (Table 5; Supplementary Table 2). One variant was considered as pathogenic and one as likely pathogenic using ACMG criteria in Varsome database, however other variants were debatable and described as likely pathogenic or VUS, similar to InterVar judgment.

**Table 5:** Summary of novel *COL4A3* variants found in a study (ARAS and ADAS).

**Variant**	**No of ind**.	**M/F**	**No of diff. families**	**Age at diagnosis (range)**	**Pro**	**KF (age at KF)**	**No of ind. with ocular ab**.	**No of ind. with hearing ab**.	**Ind. with kidney biopsy**	**ACMG (ClinVar, Varsome, InterVar, Centogene, Franklin DB)**	**Our clinical interpretation on disease course**
**One heterozygous novel variant in** ***COL4A3*** **(ADAS)**											
c.520G>A (p.Gly174Arg)	2	M-1; F-1	1	6–35	+	N/a	None	None	1 (lamellation of GBM)	3-P; 2-VUS	Likely pathogenic
c.2711G>T (p.Gly904Val)	1	M	1	31	+	N/a	None	None	1 (TBM)	1-N/A; 4-VUS	Likely benign
c.416G>A (p.Gly139Glu)	1	F	1	63	+	N/a	N/d	None	1 FSGS	1-N/A; 4-VUS	Likely pathogenic
c.4717G>A (p.Gly1573Ser)	2	M-1; F-1	1	1–30	+	N/a	1	N/d	N/a	1-N/A; 4-VUS	Likely benign or late onset
c.593G>T (p.Gly198Val)	1	M	1	53	+	N/a	None	1	1 (FSGS)	1-N/A; 4-VUS	Likely pathogenic
c.2188G>C (p.Gly730Arg)	1	F	1	42	+	N/a	None	None	1 (TBM)	1-N/A; 4-VUS	Likely benign
**Compound heterozygous variants in** ***COL4A3*** **(ARAS)**											
c.4702C>T (p.Pro1568Ser)	2	F-2	1 (twin sisters)	33	+	1	None	2	2 (FSGS)	1-N/A; 4-VUS 1-LP	Pathogenic
c.3247G>C (p.Gly1083Arg)									1-N/A; 3-VUS

*No, number; F, female; M, male; diff, different; KF, kidney failure; Ind, individual; ab., abnormalities; N/a, not applicable; N/d, no data; DIG, digenic; CKD, chronic kidney disease; FSGS, focal segmental glomerulosclerosis; pro, proteinuria; P, pathogenic; LP, likely pathogenic; VUS, variant with unknown significance; XLAS, X-linked Alport syndrome, AS, Alport syndrome, ARAS, autosomal recessive Alport syndrome; ADAS, autosomal dominant Alport syndrome*.

Of the nine different *COL4A3* variants found in the study cohort, two (78%) were glycine substitutions and two (22 %) were other missense changes (Table 5; Supplementary Table 2).

Their clinical features are seen in Table 5 and Supplementary Table 2, while one person with a digenic change is described in digenic section. Proteinuria was present in eight individuals with *COL4A3* variants. One person had reached KF due to compound heterozygous *COL4A3* variants at the age of 33. Bilateral neurosensory hearing loss and ocular lesions were found in three and one individuals, respectively.

Our clinical interpretation of novel *COL4A3* variants was based on clinical course, family history, results of kidney biopsy, sex and age; and was listed in Table 5.

### COL4A4

Twenty individuals from 11 different families had novel variants in *COL4A4*. Summary of novel *COL4A3* variants found in a study can be seen in Table 6 and in more details in Supplementary Table 3. Sixteen with AS (80 %) were carriers of only one heterozygous variant, two individuals had two heterozygous variants in *COL4A4*, and two had digenic variants in genes *COL4A4* and *COL4A5*. In total, nine novel or previously undescribed variants were identified in *COL4A4* (see Table 6; Supplementary Table 3). According to Centogene laboratory, four of these variants were classified as likely pathogenic (two of these changes were determined as probably damaging by PolyPhen-2 or disease-causing variants by MutationTester). Two novel VUS sequence variants were classified as polymorphism by MutationTaster and benign by PolyPhen-2, while one VUS was described as disease causing by both prediction programs. One variant was described as pathogenic. To compare, Franklin genetic databases described one variant as pathogenic, two variants as likely pathogenic and five variants as VUS (see Table 6; Supplementary Table 3). Two variants were considered as pathogenic and three as VUS using ACMG criteria in Varsome database, however other variants were debatable and described as likely pathogenic or VUS, although InterVar judged these variants as VUS.

**Table 6:** Summary of novel *COL4A4* variants found in a study (ARAS and ADAS).

**Variant**	**No of ind**.	**M/F**	**No of diff. families**	**Age at diagnosis (range)**	**Pro**	**KF (age at KF)**	**No of ind. with ocular ab**.	**No of ind. with hearing ab**.	**Ind. with kidney biopsy**	**ACMG (ClinVar, Varsome, InterVar, Centogene, Franklin DB)**	**Our clinical interpretation on disease course**
**One heterozygous novel variant in** ***COL4A4*** **(ADAS)**											
c.4151C>T (p.Ala1384Val)	1	F	1	31	N/d	N/a	None	None	N/a	1-N/A; 4-VUS	Likely benign
c.594+1G>A	5	M-2; F-3	2	11–64	+	1	2	1	1 (FSGS)	1-LP; 3-P	Likely pathogenic
c.2756A>G (p.Glu919Gly)	2	M-1; F-1	1	2–40	+	N/a	None	None	1 (FSGS)	5-VUS	Likely pathogenic or late onset
c.4315G>A (p.Gly1439Ser)	3	M-2; F-1	1	10–46	+	N/a	None	1	N/a	1-LP; 1-N/A; 3-VUS	Likely benign or late onset
c.3044G>A (Gly1015Glu)	3	M-3	1	2–39	None	N/a	2	N/d	N/a	2-LP; 1-N/A; 2-VUS	Likely benign
c.657+2dup	1	F	1	65	None	N/a	None	1	1 (TBM)	1-LP; 2-N/A; 2-VUS	Likely benign
c.2347G>A (p.Gly783Arg)	1	F	1	4	None	N/a	None	None	N/a	1-LP; 4-VUS	Likely benign Difficult to interpret due to young age of individual
**Compound heterozygous variants in** ***COL4A4*** **(ARAS)**											
c.594+1G>A (novel)	1	M	1	6	+	N/a	None	None	1	LP; 3-P	Pathogenic
p.Gly527Cys (described variant)									LP
c.4720C>T (p.Gln1574*) (novel)	1	F	1	32	+	N/a	None	None	1 (TBM)	LP; 3-P; 1-N/A	Likely benign (eGFR normal)
c.3307G>A (p.Gly1103Arg) (described variant)									LP

*No, number; F, female; M, male; diff, different; KF, kidney failure; Ind, individual; ab., abnormalities; N/a, not applicable; N/d, no data; DIG, digenic; CKD, chronic kidney disease; FSGS, focal segmental glomerulosclerosis; pro, proteinuria; P, pathogenic; LP, likely pathogenic; VUS, variant with unknown significance; XLAS, X-linked Alport syndrome, AS, Alport syndrome, ARAS, autosomal recessive Alport syndrome; ADAS, autosomal dominant Alport syndrome*.

Of the nine identified different *COL4A4* variants, six variants were missense changes of which 3 (33%) were glycine substitutions (Table 6; Supplementary Table 3), while the remaining three variants were classified as other missense variants. One splice site, and one stop codon changes detected in the *COL4A4* gene are presented in Table 6 and Supplementary Table 3. The analysis of the pathogenic potential of these sequence variants by Human Splicing Finder MutationTaster, suggested functional loss of the damaged protein caused by aberrant splicing or stop codon.

The clinical features are seen in Table 6, while two individuals with digenic variants are described in digenic section. Proteinuria was discovered in eight of 18 individuals. One person with heterozygous *COL4A4* variant reached KF at the age 55, while eGFR (estimated glomerular filtration rate, using CKD-EPI, Chronic Kidney Disease Epidemiology Collaboration formula) <30 ml/min/1.73 m^2^ was revealed in 11% (2/18) of individuals with *COL4A4* changes. Bilateral neurosensory hearing loss and ocular lesions were found in 3 and 4 individuals, respectively.

Our clinical interpretation of novel *COL4A4* variants was based on clinical course, family history, results of kidney biopsy, sex and age; and was listed in Table 6.

### Digenic

Three individuals from three different families carried digenic variants. Genetic analysis indicated a frameshift change in the *COL4A5* gene combined with a missense variant in the *COL4A4* gene in 2 individuals (Table 7; Supplementary Tables 1, 3). One person had one novel missense change in *COL4A3* and a previously reported in the literature splice site variant in *COL4A4* (Table 7; Supplementary Table 2). Altogether, five novel digenic variants were identified. Two novel changes in *COL4A4* (33%) were classified as VUS. These two variants p.Arg1637Gln and p.Ala1384Val in *COL4A4* were described as probably damaging and benign, respectively, by both prediction tools (PolyPhen-2, and MutationTaster). Two identified variants in *COL4A5*, according to Centogene, were classified as pathogenic, whereas N/a by PolyPhen-2, and MutationTaster. One novel variant in *COL4A3* p.Arg341Cys was judged as disease causing by one program and polymorphism by the other.

**Table 7:** Summary of novel digenic variants in *COL4A3-A5* (digenic AS).

**Variant**	**No of ind**.	**M/F**	**No of diff. families**	**Age at diagnosis (range)**	**Pro**	**KF (age at KF)**	**No of ind. with ocular ab**.	**No of ind. with hearing ab**.	**Ind. with kidney biopsy**	**ACMG (ClinVar, Varsome, InterVar, Centogene, Franklin DB)**	**Our clinical interpretation on disease course**
**Digenic variants in** ***COL4A5*** **and** ***COL4A4***											
*COL4A5* c.1417_1418del (p.Val473Glufs*3)	1	F	1	14	+	N/a	None	None	1 (FSGS)	3-LP; 1-P; 1-N/A	Likely pathogenic
*COL4A4* c.4151C>T (p.Ala1384Val)									1-N/A; 4-VUS
*COL4A5* c.1374delinsTT(p.Pro459Serfs*6)	1	F	1	26	+	N/a	None	None	N/a	3-LP; 1-P; 1-N/A	Likely benign
*COL4A4* c.4910G>A (p.Arg1637Gln)									1-N/A; 4-VUS
**Digenic variants in** ***COL4A3*** **and** ***COL4A4***											
*COL4A3* c.1021C>T (p.Arg341Cys)	1	M	1	41	+	N/a	None	None	1 (TBM)	1-LP; 1-P; 1-N/A; 2-VUS	Likely benign
*COL4A4* c.-101-4A>G									Polymorphism

*No, number; F, female; M, male; diff, different; KF, kidney failure; Ind, individual; ab., abnormalities; N/a, not applicable; N/d, no data; DIG, digenic; CKD, chronic kidney disease; FSGS, focal segmental glomerulosclerosis; pro, proteinuria; P, pathogenic; LP, likely pathogenic; VUS, variant with unknown significance; XLAS, X-linked Alport syndrome, AS, Alport syndrome, ARAS, autosomal recessive Alport syndrome; ADAS, autosomal dominant Alport syndrome*.

Both identified variants in *COL4A4* were missense variants, while variants in *COL4A5* were associated with frame shift changes (Table 7; Supplementary Tables 1, 3). One glycine substitution was discovered in *COL4A3* (Table 7; Supplementary Table 2). All individuals had proteinuria, neither of them had a neurosensory hearing loss nor ocular lesion. None of the individuals reached KF.

Our clinical interpretation of novel digenic variants was based on clinical course, family history, results of kidney biopsy, sex and age; and was listed in Table 7.

## Discussion

Heterozygous disease causing variants in *COL4A3* or *COL4A4* demonstrate wide phenotypic spectrum of manifestations, ranging from asymptomatic isolated hematuria to progressive kidney disease and extrarenal lesions (13). In cases with isolated hematuria, the terms “thin basement membrane disease,” “familial benign hematuria,” and “carriers of ARAS” have been used (17–19). However, many individuals with heterozygous *COL4A3* or *COL4A4* disease causing variants eventually develop FSGS and severe proteinuria and some progress to KF (20, 21). The aim of redefining nomenclature from TBMN to ADAS is to simplify the diagnostic terminology and to improve the early diagnosis of AS and increase the chances of affected individuals receiving proper monitoring and well-timed therapy (13). Therefore, in this study we used ADAS terminology for the heterozygous *COL4A3* and *COL4A4* variants. According to Kashtan, 1% of individuals with ADAS reach KF, while this increases in those with risk factors such as proteinuria, FSGS, GBM thickening and lamellation, family history of progressive kidney disease, up to 20% of cases (13). In our study, one person out of 24 developed KF at the age of 55 years (A49737, COL4A4 c.594+1G>A; NM_000092.5). In a similar study, individuals with ADAS reached KF at a median age of 70 years (22). The ADAS burden depends on the type of pathogenic change. Nonsense and stop codon variants are more often associated with KF compared with missense variants (9). In our study, the person who developed KF had a splice site variant in *COL4A4*. There were also other risk factors for KF such as FSGS in the kidney biopsy, severe proteinuria, and late diagnosis of AS, with no early initiation of renoprotective treatment. However, the individual's offspring, only had persistent hematuria and minimal proteinuria even as adults. Interestingly, one person's grandson (A4/49716^3*bi^) was diagnosed with ARAS due to compound heterozygous variants in the *COL4A4* gene, inherited from his mother (the person's daughter) and his father. The same variant (*COL4A4* c.594+1G>A; NM_000092.5) was also found in 64-year-old male (A494487) from a different, unrelated family, who at the time of diagnosis, had chronic kidney disease (CKD) stage IV and his kidney histology revealed FSGS. Despite severe kidney function impairment, the proteinuria level was low (<1 g/d). Therefore, the same variant can be associated with interfamilial and extrafamilial variation and emphasizes the importance of careful evaluation and monitoring of individuals with heterozygous *COL4A3* or *COL4A4* variants. Although extrarenal manifestations in ADAS are rare, one study reported that 13.3% of individuals with ADAS developed a sensorineural hearing loss (23). However, these results are consistent with our study. Another study reported that 23% of individuals had abnormal ocular findings, but only two individuals (<1%) had anomalies possibly related to AS (24). In our study, ocular abnormalities, including retinal flecks and retinal thinning, were found in 25% of individuals with heterozygous *COL4A3* and *COL4A4* variants. These results indicate that phenotypic manifestations in individuals with ADAS vary significantly. However, extrarenal expression is usually milder and appears less frequently than in XLAS males and ARAS individuals. Nevertheless, the development of risk factors may have a significant impact on the disease burden (3, 25). Alternatively our individuals with hearing loss and ocular abnormalities could have had a second undetected pathogenic variant and hence ARAS or digenic AS.

Genotype-phenotype correlations are only now emerging in ARAS individuals (26). One study reported that ARAS individuals with nonsense variants reach KF earlier in life and frequently have more nephrotic range proteinuria with a higher prevalence of ocular and sensorineural hearing changes, than with compound or biallelic missense variants (27). In our study, KF was found in only one woman (A3/3232145^5*bi^) at the age of 33 with compound novel heterozygous missense variants c.4702C>T (p.Pro1568Ser) and c.3247G>C (p.Gly1083Arg) in *COL4A3*. Her biopsy demonstrated FSGS and a thinned and lamellated glomerulus basement membrane. Interestingly, the person's heterozygous twin sister (A3/3232146^5*bi^) with the same variants, had a milder clinical phenotype but similar kidney biopsy features including FSGS. Different phenotypes might be explained by the dizygotic form of the twins and modifying environmental factors, and more Alport twin studies might be helpful. One study reported that hematuria and proteinuria were found in all individuals with ARAS and 43% of them developed KF (26). Both twins in our study had nephrotic range proteinuria which is a prognostic factor for KF. The third person ($A4/49716^{3{*}^{\text{bi}}}$) with ARAS, diagnosed at the age of 6, was from a different family and had two compound heterozygous *COL4A4* variants gene. A novel pathogenic splice site variant c.594+1G>A (NM_000092.5) was detected in *COL4A4* and shared its phenotype with the already described likely pathogenic variant in *COL4A4* c.1579G>T (p.Gly527Cys) (22). This boy (A4/49716^3*bi^) at the age of 12 had both hematuria and proteinuria (detected from the age of 1), without any deterioration of kidney function. Many cases of ARAS are overlooked because of the lack of a positive family history and different genotype-phenotype correlations among family members (28).

Large fragment deletions, nonsense variants or frameshift variants are associated with a higher risk of KF than splicing or missense variants in *COL4A5* gene (12), but in this study the most severe phenotype including KF was associated with a missense *COL4A5* variant c.2777G>T (p.Gly926Val, NM_033380.3). This novel *COL4A5* variant causes an amino acid change from Gly to Val at position 926 and is classified as a variant of uncertain significance in Centogene and Franklin's databases, while Varsome database describes this variant as likely pathogenic (PM2, MM1, PP2, PP3). Two brothers from the same family with this variant developed KF by 24 and 32 years of age. Interestingly the younger individual developed KF 8 years earlier than his older brother, suggesting that the same gene variants might result in significantly different phenotypes. In individuals with AS, the variant type determines the age at KF in general (29). In our study, only two of seven males (29%) with XLAS developed KF, which is consistent with a better prognosis than noted previously (29% compared with 90%) (10). The mean age of KF in individuals with missense variants, splicing site variants, and truncation variants have been reported as 37, 28, and 25 years, respectively (8). Males studied here were relatively young (median age of 24 years), at the time of diagnosis and evaluation of the phenotype, which might explain the lower rate of KF than in other studies. However, four individuals with *COL4A5* variants had significant nephrotic range proteinuria (≥3, 5 g/d) which can also be associated with a future decline in kidney function and the main predictor of KF (30–32).

The phenotype in women is usually milder (10, 33), however it is known that heterozygous females with XLAS still have a lifetime risk of KF and other extrarenal manifestations (34). Although in our study most of the females had only hematuria, three women A5338, A56511, A5343159 with heterozygous variants p.Gly1170Arg, Val473Glufs^*^3, p.Gly156Arg in *COL4A5* had decline of eGFR < 60 ml/min/1.73 m^2^. A genotype - phenotype correlations demonstrate a high intrafamilial heterogeneity due to random X-chromosomal inactivation thus prediction of the disease course is complicated (12). Bilateral sensorineural hearing loss appeared in 71.4% individuals with XLAS, which consists with other studies (10, 35). According to several studies, lenticonus may have a prognostic significance on eGFR decline (10). In our study, two males with anterior lenticonus presented with KF. However, further studies are required to address the exact relationship between kidney and extrarenal manifestations.

Digenic inheritance still raises many questions, and a more complex inheritance should be considered when reviewing prognosis and at-risk family members (36). In our study three individuals aged 14–41, from three different families, had digenic variants in these genes. According to the recent guidelines for Genetic Testing and Management of Alport Syndrome, digenic inheritance may be associated with more severe phenotypes including kidney impairment (37). However, in our study none of individuals with digenic variants regardless of the variant type (missense and splicing changes) reached KF. Variants in the *COL4A5* gene in combination with *COL4A3* or *COL4A4* variants have on-Mendelian inheritance and therefore, family-specific risk assessment is critical.

## Conclusion

In conclusion, 27 novel pathogenic variants were identified by NGS testing of all three *COL4A3-A5* genes in individuals with suspected AS from a single center over a 5-year period. All index cases had hematuria and four (7.8%) had KF. About half the affected individuals had ADAS but this cohort also included ARAS, XLAS and digenic disease. These novel variants represented more than half of all the variants found in a cohort of 171 individuals from 109 unrelated families who underwent testing. We identified pathogenic or likely pathogenic variants in all the individuals with KF or with ocular abnormalities consistent with a high detection rate in XL and AR Alport syndrome. The detection rate of pathogenic variants was less in those with persistent hematuria alone. These results increase the number of known *COL4A3, COL4A4*, and *COL4A5* variants and our understanding of genotype-phenotype correlations in AS. The genetic variants in *COL4A3-5* have a significant impact on the diagnosis, treatment, and prognosis for individuals with AS.

## Data Availability Statement

The original contributions presented in the study are publicly available. This data can be found here: ClinVar Database, accession SUB11112191 (SCV002098105 - SCV002098129). However, authors declare that in this study there is a partial non-availability of raw data due to the time period restriction imposed by Centogene laboratory on stored data (older than 5 years variants and its raw data are not stored in this laboratory). Data non-availability could potentially be a major limitation of the study.

## Ethics Statement

The studies involving human participants were reviewed and approved by the Vilnius Regional Biomedical Research Ethics Committee of Lithuania (BioAlport, No 158200-16-857-367). Written informed consent to participate in this study was provided by the participants' legal guardian/next of kin. Written informed consent was obtained from the individual(s), and minor(s)' legal guardian/next of kin, for the publication of any potentially identifiable images or data included in this article.

## Author Contributions

AC, RC, and JS contributed to conception and design of the study. AC, RS-S, VV, RC, BB, AJ, AL, MM, PB, SS, and AR organized the database. AC performed the statistical analysis and wrote the first draft of the manuscript. AC, KJ, IJ, RC, and JS wrote sections of the manuscript. All authors contributed to manuscript revision, read, and approved the submitted version.

## Conflict of Interest

AR, PB, and SS were employed by CENTOGENE. The genetic testing for the individuals of this research was made by CENTOGENE free of charge by participating in other research - BioAlport study. The remaining authors declare that the research was conducted in the absence of any commercial or financial relationships that could be construed as a potential conflict of interest.

## Publisher's Note

All claims expressed in this article are solely those of the authors and do not necessarily represent those of their affiliated organizations, or those of the publisher, the editors and the reviewers. Any product that may be evaluated in this article, or claim that may be made by its manufacturer, is not guaranteed or endorsed by the publisher.
