# Supplementary material for: Identification of 27 Novel Variants in Genes COL4A3, COL4A4, and COL4A5 in Lithuanian Families With Alport Syndrome
Source: Front Med (Lausanne). 2022 Mar 28;9:859521. doi: 10.3389/fmed.2022.859521 (PMC8995700; doi:10.3389/fmed.2022.859521)
Supplement: Supplementary file 1 [file Data_Sheet_1.pdf]

Suppl. Table 1. Characteristics of 9 novel *COL4A5* variants in 22 individuals with XLAS or digenic AS: genotype – phenotype

Patient ID	Var No	Family	Family Mem	Age at diagnosis	M/F	Variant coordinates	Nucleotide	Protein change	Type of variant	ClinVar DB	ACMG (InterVar)	ACMG (Varsome)	ACMG (Centogene)	ACMG (Franklin)	Heterozygosity	PP2	SIFT	MT	Align-GVGD
***COL4A5* gene**																			
A5336	1	1	I	11	F	NM_033380.2	c.3508G>C	p.Gly1170Arg	missense	N/a	LP	P	LP	LP	Het	pd	pd	pd	C65
A5337			II	43	F										Het				
A5338			III	17	M										Hem				
A5449	2	2	I	10	M	NM_033380.2	c.3106+1G>A	-	splice site	N/a	P	P	LP	LP	Hemi	N/a	N/a	N/a	N/a
A54410			II	4	F										Het				
A54490			III	37	F										Het				
A56511	3	3	I	36	F	NM_033380.1	c.1417_1418del	p.Val473Glufs*3	frameshift	N/a	LP	LP	P	LP	Het	N/a	N/a	N/a	N/a
A5/46512[1*] DIG			II[1*]	14	F										Het				
A56513			III	33	F										Het				
A56514			IV	28	F										Het				
A5141023	4	4	I	6	M	NM_033380.	c.347delC	p.Pro116Glnfs*39	frameshift	N/a	P	LP	P	LP	Hemi	N/a	N/a	N/a	N/a
A5141024			II	35	F										Het				
A5181635	5	5	I	22	M	NM_033380.2	c.883G>A	p.Gly295Ser	missense	N/a	VUS	P	VUS	LP	Hemi	N/a	N/a	N/a	N/a
A5211939	6	6	I	28	M	NM_033380.3	c.2777G>T	p.Gly926Val	missense	N/a	VUS	LP	VUS	VUS	Hemi	N/a	N/a	N/a	N/a
A5211940			II	24	M										Hemi				
A5211941			III	47	F										Het				
A5/4262450 [2*DIG]	7	7	I[2*]	26	F	NM_033380.1	c.1374delinsTT	p.Pro459Serfs*6	frameshift	N/a	LP	LP	P	LP	Het	N/a	N/a	N/a	N/a
A5262451			II	20	M										Hemi				
A5262452			III	51	F										Het				
A5333058	8	8	I	9	F	NM_033380.1	c.3554-2A>G	-	splice site	N/a	P	P	LP	LP	Het	dc	N/a	N/a	N/a
A5333078			II	14	F										Het				
A5343159	9	9	I	15	F	NM_033380.1	c.466G>C	p.Gly156Arg	missense	N/a	VUS	P	VUS	VUS	Het	pd	dlt	dc	C65

Patient ID	Proteinuria (Age at onset)	Proteinuria (amount)	CKD stage*	KF (age at onset)	Kidney biopsy (Age at biopsy)	Ocular abnormalities	Hearing abnormalities	Comments
A5336	6	+	I	N/a	N/a	None	None	
A5337	25	++	II	N/a	N/a	None	None	AH+ ACE inhibitors
A5338	7	+++	IIIb	N/a	+ (16)<br>FSGS, tubular vacuolization and atrophy, interstitial foam cells, thinning, thickening, lamellation of GBM, foot process effacement	None	None	AH+ ACE inhibitors + ARB
A5449	9	+	I	N/a	N/a	+	+	ACE inhibitors
A54410	None	Normal	I	N/a	N/a	None	None	
A54490	37	+	II	N/a	N/a	None	None	ACE inhibitors
A56511	21	+	IIIa	N/a	N/a	None	+	AH + ACE inhibitors
A5/46512[1*DIG]	14	+	I	N/a	+ (14)<br>FSGS, tubular vacuolization and atrophy, thinning, thickening, lamellation of GBM, foot process effacement	None	None	Digenic AS. This individual has two het variants and shares phenotype with other novel *COL4A4* variant p.Ala1384Val. Please see Table 4, variant 19; ID A5/46512[1*DIG]
A56513	None	Normal	I	N/a	N/a	None	None	
A56514	32	+	I	N/a	N/a	None	None	
A5141023	3	++	I	N/a	+ (6)<br>Thinning, thickening, lamellation of GBM, foot process effacement	N/d	+	
A5141024	None	Normal	I	N/a	N/a	None	None	
A5181635	13	++	I	N/a	+ (22)<br>Tubular vacuolization, thinning, thickening, lamellation of GBM, foot process effacement	None	None	
A5211939	6	+++	V	+ (32)	+ (20)<br>Interstitial foam cells, tubular atrophy	+	+	
A5211940	2	+++	V	+ (24)	N/a	+	+	Renal Tx
A5211941	19	+	I	N/a	N/a	None	None	
A5/4262450[2*DIG]	16	+	I	N/a	N/a	None	None	Digenic AS. This individual has two het variants and shares phenotype with other novel *COL4A4* variant p.Arg1637Gln. Please see Table 4, variant 22; ID A5/4262450[2*DIG]
A5262451	6	++	I	N/a	N/a	+	+	
A5262452	18	+	I	N/a	N/a	None	None	
A5333058	7	+	I	N/a	+ (9)<br>FSGS, tubular vacuolization and atrophy, thinning, irregular contour of GBM, foot process effacement	N/d	N/d	
A5333078	12	+	I	N/a	+ (12)<br>FSGS, tubular vacuolization and atrophy, thinning, irregular contour of GBM, foot process effacement	N/d	N/d	
A5343159	9	+++	IIIb	N/a	+ (9)<br>FSGS, tubular vacuolization and atrophy, thinning, lamellation of GBM, foot process effacement	None	None	AH + ACE inhibitors

AlignGVD: C0: least likely to interfere with function, C65: most likely to interfere with function; Gly subs – glycine substitution; Hemi- hemizygous; Het – heterozygous; pd-probably damaging; dlt – deleterious; dc – disease causing; plm – polymorphism; bn – benign variant; tlt – tolerated; pat – pathological; N/a – not applicable; chr – chromosome; DIG – digenic; bi – biallelic; cis/trans – unknown position; mem – member; F – female; M – male.; PP2 – PolyPhen-2; MT – MutationTester; DB – database; Cento – Centogene; var – variant; P- pathogenic; LP – likely pathogenic; VUS – variant with unknown significance

KF- kidney failure; DIG – digenic; bi – biallelic; CKD – chronic kidney disease; MA – microalbuminuria; FSGS – focal segmental glomerulosclerosis; AH – arterial hypertension; ACE inhibitors – angiotensin converting enzyme inhibitors; mem – member; Tx – transplantation; AR- autosomal recessive inheritance; N/d – no data; XLAS – X-linked Alport syndrome, AS – Alport syndrome, ARAS – autosomal recessive Alport syndrome
*CKD stages are classified based on eGFR (estimated glomerular filtration rate, using EPI-CKD formula) as follows: stage I (eGFR >90 mL/min/1.73 m2); stage II (eGFR 60-89 mL/min/1.73 m2); stage IIIa (eGFR 45-59 mL/min/1.73 m2); stage IIIb (eGFR 30-44 mL/min/1.73 m2); stage IV (eGFR 15-29 mL/min/1.73 m2); stage V (eGFR < 15 mL/min/1.73 m2)

Suppl. Table 2. Characteristics of 9 novel *COL4A3* variants in 11 individuals (with heterozygous variants, digenic AS or ARAS): genotype – phenotype

Patient ID	Var No	Family	Family Mem	Age at diagnosis	M/F	Variant coordinates	Nucleotide	Protein change	Type of variant	ClinVar DB	ACMG (InterVar)	ACMG (Varsome)	ACMG (Centogene)	ACMG (Franklin)	Heterozygosity	PP2	SIFT	MT	Align-GVGD
***COL4A3* gene**																			
A3191736	10	10	I	35	F	NM_000091.4	c.520G>A	p.Gly174Arg	missense	P	VUS	P	VUS	P	Het	pd	dlt	dc	C0
A3191774			II	6	M										Het				
A3/3232145[5*] bi	11	11	I	33	F	NM_000091.3	c.4702C>T	p.Pro1568Ser	missense	N/a	VUS	VUS	VUS	VUS	Het	pd	dlt	dc	C65
A3/3232146[5*] bi			II	33	F										Het				
A3/3232145[5*] bi	12	11	I	33	F	NM_000091.3	c.3247G>C	p.Gly1083Arg	missense	N/a	VUS	LP	VUS	VUS	Het	pd	dlt	dc	C0
A3/3232146[5*] bi			II	33	F										Het				
A3312856	13	12	I	31	M	NM_000091.3 Exon 33	c.2711G>T	p.Gly904Val	missense	N/a	VUS	VUS/LP	VUS	VUS	Het	pd	dlt	dc	C0
A3373665	14	13	I	63	F	NM_000091.3	c.416G>A	p.Gly139Glu	missense	N/a	VUS	VUS/LP	VUS	VUS	Het	pd	dlt	dc	C15
A3/4383766[6*] DIG	15	14	I	41	M	NM_000091.3	c.1021C>T	p.Arg341Cys	missense	N/a	VUS	VUS/LP	VUS	VUS	Het	pd	dlt	plm	C15
A3353268	16	15	I	1	F	NM_000091.3	c.4717G>A	p.Gly1573Ser	missense	N/a	VUS	VUS/LP	VUS	VUS	Het	-	dlt	dc	C0
A3353296			II	30	M										Het				
A3394081	17	16	I	53	M	NM_000091.3	c.593G>T	p.Gly198Val	missense	N/a	VUS	VUS/LP	VUS	VUS	Het	pd	dlt	dc	C65
A3435095	18	17	I	42	F	NM_000091.3	c.2188G>C	p.Gly730Arg	missense	N/a	VUS	VUS/LP	VUS	VUS	Het	-	dlt	dc	C0

Patient ID	Proteinuria (Age at onset)	Proteinuria (Amount)	CKD stage*	KF (Age at onset)	Renal biopsy (Age at biopsy)	Ocular abnormalities	Hearing abnormalities	Comments
A3191736	29	++	II	N/a	+ (33)<br>GBM thinning, thickening, lamellation, foot process effacement	None	None	
A3191774	None	Normal	I	N/a	N/a	None	None	
A3/3232145[5*bi]	23	+++	V	33	+ (31)<br>FSGS	None	+	ARAS (compound 2 het variants). This individual has two het variants and shares phenotype with two novel het variants p.Pro1568Ser (variant 11) and p.Gly1083Arg (variant 12) in COL4A3 gene.<br>Twin sister of A3/3232146[5*bi]
A3/3232146[5*bi]	30	++	IIIb	N/a	+ (32)<br>FSGS, tubular vacuolization and atrophy, GBM thinning, thickening lamellation, foot process effacement	None	+	ARAS (compound 2 het variants). This individual has two het variants and shares phenotype with two novel het variants p.Pro1568Ser (variant 11) and p.Gly1083Arg (variant 12) in COL4A3 gene. Twin sister of A3/3232145[5*bi]
A3312856	15	+	I	N/a	+ (30)<br>Tubular vacuolization, thinning of GBM, foot process effacement	None	None	
A3373665	None	Normal	II	N/a	+ (62)<br>FSGS, tubular vacuolization, fibrosis, atrophy, thinning of GBM, foot process effacement, positive for IgA, IgG, IgM and C3.	N/d	None	
A3/4383766[6*DIG]	40	+	I	N/a	+ (40)<br>Tubular vacuolization, thinning of GBM, foot process effacement	None	None	Digenic AS. This individual has two het variants and shares phenotype with other variant c.-101-4A>G in *COL4A4* (polymorphism in ClinVar).
A3353268	1	+	I	N/a	N/a	+	None	
A3353296	N/d	N/d	N/d	N/a	N/a	N/d	N/d	
A3394081	44	+	I	N/a	+ (53)<br>FSGS, tubular vacuolization, atrophy, fibrosis, thinning of GBM, foot process effacement	None	+	
A3435095	42	++	I	N/a	+ (43)<br>Tubular vacuolization, atrophy, thinning of GBM, foot process effacement	None	None	

AlignGVD: C0: least likely to interfere with function, C65: most likely to interfere with function; Gly subs – glycine substitution; Hemi- hemizygous; Het – heterozygous; pd-probably damaging; dlt – deleterious; dc – disease causing; plm – polymorphism; bn – benign variant; tlt – tolerated; pat – pathological; N/a – not applicable; chr – chromosome; DIG – digenic; bi – biallelic; cis/trans – unknown position; mem – member; F – female; M – male.; PP2 – PolyPhen-2; MT – MutationTester; DB – database; Cento – Centogene; var – variant; P- pathogenic; LP – likely pathogenic; VUS – variant with unknown significance

KF- kidney failure; DIG – digenic; bi – biallelic; CKD – chronic kidney disease; MA – microalbuminuria; FSGS – focal segmental glomerulosclerosis; AH – arterial hypertension; ACE inhibitors – angiotensin converting enzyme inhibitors; mem – member; Tx – transplantation; AR- autosomal recessive inheritance; N/d – no data; XLAS – X-linked Alport syndrome, AS – Alport syndrome, ARAS – autosomal recessive Alport syndrome
*CKD stages are classified based on eGFR (estimated glomerular filtration rate, using EPI-CKD formula) as follows: stage I (eGFR >90 mL/min/1.73 m2); stage II (eGFR 60-89 mL/min/1.73 m2); stage IIIa (eGFR 45-59 mL/min/1.73 m2); stage IIIb (eGFR 30-44 mL/min/1.73 m2); stage IV (eGFR 15-29 mL/min/1.73 m2); stage V (eGFR < 15 mL/min/1.73 m2)

Suppl. Table 3. Characteristics of 9 novel *COL4A4* variants in 20 individuals (with heterozygous variants, digenic AS or ARAS): genotype – phenotype

Patient ID	Var No	Family	Family Mem	Age at diagnosis	M/F	Variant coordinates	Nucleotide	Protein change	Type of variant	ClinVar DB	ACMG (InterVar)	ACMG Varsome)	ACMG (Centogene)	ACMG (Franklin)	Heterozygosity	PP2	SIFT	MT	Align-GVGD
***COL4A4* gene**																			
A5/46512[1*DIG]	19	3	I	14	F	NM_000092.4	c.4151C>T	p.Ala1384Val	missense	N/a	VUS	VUS	VUS	VUS	Het	bng	tlt	plm	C0
A475197		18	I	31	F										Het				
A4/49716[3*bi]	20	19	I	6	M	NM_000092.5	c.594+1G>A	-	splice site	LP	P	P	LP	P	Het[*bi]	N/a	N/a	N/a	N/a
A49717			II	34	F										Het				
A49737			III	64	F										Het				
A49775			IV	11	F										Het				
A49738			V	30	M										Het				
A494487		20	I	64	M										Het				
A4/4161430[4* cis/trans]	21	21	I	32	F	NM_000092.4	c.4720C>T	p.Gln1574*	stop codon	N/a	P	P	P	LP	Het	N/a	N/a	N/a	N/a
A5/4262450[2* DIG]	22	7	I	26	F	NM_000092.4	c.4910G>A	p.Arg1637Gln	missense	N/a	VUS	VUS	VUS	VUS	Het	pd	tlt	dc	C0
A4322957	23	22	I	40	F	NM_000092.4	c.2756A>G	p.Glu919Gly	missense	VUS	VUS	VUS	VUS	VUS	Het	bn	dlt	plm	C0
A4322976			II	2	M										Het				
A4363463	24	23	I	20	M	NM_000092.4	c.4315G>A	p.Gly1439Ser	missense	N/a	VUS	VUS/LP	LP	VUS	Het	pd	dlt	dc	C0
A4363469			II	46	F										Het				
A4363477			III	10	M										Het				
A4404182	25	24	I	39	M	NM_000092.5	c.3044G>A	Gly1015Glu	missense	LP or VUS	VUS	VUS/LP	N/a	LP	Het	pat	pat	pat	N/a
A4404185			II	4	M										Het				
A4404186			III	2	M										Het				
A453971	26	25	I	65	F	NM_000092.4	c.657+2dup	-	unknown	N/a	VUS	VUS	LP	N/a	Het	N/a	N/a	N/a	N/a
A4414384	27	26	I	4	F	NM_000092.4	c.2347G>A	p.Gly783Arg	missense	VUS	VUS	VUS/LP	LP	VUS	Het	pd	dlt	dc	C15

Patient ID	Proteinuria (Age at onset)	Proteinuria (amount)	CKD stage*	KF (Age at onset)	Renal biopsy (Age at biopsy)	Ocular abnormalities	Hearing abnormalities	Other comments
A5/46512[1*DIG]	14	+	I	N/a	+ (14)<br>FSGS, tubular vacuolization, atrophy; GBM thinning, thickening, lamellation; foot process effacement	None	None	Digenic AS. This individual has two het variants and shares phenotype with other novel het variant p.Val473Glufs*3 in COL4A5. Please see Table 2. Var No3, ID A5/46512[1*DIG]
A475197	N/d	N/d	N/d	N/a	N/d	None	None	
A4/49716[3*bi]	9 months	++	I	N/a	+ (5)<br>Data is not available	None	None	ACE inhibitors<br>ARAS (compound het). This individual has two het variants and shares phenotype with other variant p.Gly527Cys in *COL4A4* (ClinVar: likely pathogenic)
A49717	29	+ (MA)	I	N/a	-	None	+	One lateral hearing abnormality
A49737	54	++	V	55	+ (54)<br>FSGS	None	None	Renal Tx
A49775	11	+	I	N/a	-	+	None	
A49738	Normal	None	I	N/a	-	+	None	
A494487	64	+	IV	N/a	+ (64)<br>Tubular vacuolization, atrophy; thinning of GBM, foot process effacement	None	None	
A4/4161430[4*cis/trans]	32	+	I	N/a	+ (32)<br>Thinning of GBM, foot process effacement	None	None	This individual has two het variants and shares phenotype with other known *COL4A4* variant p.Gly1103Arg (ClinVar: likely pathogenic). No availability of parents' screening.
A5/4262450[2*DIG]	16	+	I	N/a	-	None	None	Digenic AS. This individual has two het variants and shares phenotype with other with novel variant p.Pro459Serfs*6 in *COL4A5*. Please see Table 2, Var No7, ID A5/4262450[2*DIG]
A4322957	30	+	I	N/a	+ (40)<br>FSGS; tubular vacuolization, atrophy; GBM thinning, lamellation; foot process effacement	None	None	
A4322976	Normal	None	I	N/a	-	None	None	
A4363463	20	+	I	N/a	-	None	None	
A4363469	Normal	None	I	N/a	-	None	+	
A4363477	Normal	None	II	N/a	-	None	None	
A4404182	Normal	None	I	N/a	-	None	None	
A4404185	Normal	None	I	N/a	-	+	N/d	Ocular abnormalities may be common for AS but inherited from patient's mother with no AS
A4404186	Normal	None	I	N/a	-	+	N/d	Ocular abnormalities may be common for AS but inherited from patient's mother with no AS
A453971	Normal	None	II	N/a	+ (65)<br>Tubular vacuolization, atrophy; thinning of GBM, foot process effacement	None	+	AH + ACE inhibitors
A4414384	Normal	None	I	N/a	-	None	None	Positive family history for CKD (patient's father and grandfather)

AlignGVD: C0: least likely to interfere with function, C65: most likely to interfere with function; Gly subs – glycine substitution; Hemi- hemizygous; Het – heterozygous; pd-probably damaging; dlt – deleterious; dc – disease causing; plm – polymorphism; bn – benign variant; tlt – tolerated; pat – pathological; N/a – not applicable; chr – chromosome; DIG – digenic; bi – biallelic; cis/trans – unknown position; mem – member; F – female; M – male.; PP2 – PolyPhen-2; MT – MutationTester; DB – database; Cento – Centogene; var – variant; P- pathogenic; LP – likely pathogenic; VUS – variant with unknown significance

KF- kidney failure; DIG – digenic; bi – biallelic; CKD – chronic kidney disease; MA – microalbuminuria; FSGS – focal segmental glomerulosclerosis; AH – arterial hypertension; ACE inhibitors – angiotensin converting enzyme inhibitors; mem – member; Tx – transplantation; AR- autosomal recessive inheritance; N/d – no data; XLAS – X-linked Alport syndrome, AS – Alport syndrome, ARAS – autosomal recessive Alport syndrome
*CKD stages are classified based on eGFR (estimated glomerular filtration rate, using EPI-CKD formula) as follows: stage I (eGFR >90 mL/min/1.73 m2); stage II (eGFR 60-89 mL/min/1.73 m2); stage IIIa (eGFR 45-59 mL/min/1.73 m2); stage IIIb (eGFR 30-44 mL/min/1.73 m2); stage IV (eGFR 15-29 mL/min/1.73 m2); stage V (eGFR < 15 mL/min/1.73 m2)
